# Identification and analysis of differential miRNA–mRNA interactions in coronary heart disease: an experimental screening approach

**DOI:** 10.3389/fcvm.2023.1186297

**Published:** 2023-10-30

**Authors:** Jie Wang, Lanchun Liu, Chao Liu, Nuo Cheng, Qiyuan Mao, Cong Chen, Jun Hu, Haoqiang He, Xiaoshan Hui, Peirong Qu, Wenjing Lian, Lian Duan, Yan Dong, Yongmei Liu, Jun Li

**Affiliations:** ^1^ Department of Cardiology, China Academy of Chinese Medical Sciences Guang'anmen Hospital, Beijing, China; ^2^ Department of Graduate, Beijing University of Chinese Medicine, Beijing, China; ^3^ Department of Oncology, China Academy of Chinese Medical Sciences Guang'anmen Hospital, Beijing, China

**Keywords:** traditional Chinese medicine, coronary heart disease, kidney deficiency and blood stasis, bioinformatics analysis, experimental validation

## Abstract

**Objective:**

This aim of this study is to screen the differential molecules of kidney deficiency and blood stasis (KDBS) syndrome in coronary heart disease by high-throughput sequencing. In addition, the study aims to verify the alterations in the expression levels of miR-4685-3p and its regulated downstream, namely, C1QC, C4, and C5, using quantitative polymerase chain reaction (qPCR) and enzyme-linked immunosorbent assay (ELISA), and to determine whether the complement and coagulation cascade pathway is the specific pathogenic pathway.

**Methods:**

Patients diagnosed with unstable angina pectoris with KDBS syndrome, patients with non-kidney deficiency blood stasis (NKDBS) syndrome, and a Normal group were recruited. The clinical symptoms of each group were further analyzed. Illumina's NextSeq 2000 sequencing platform and FastQC software were used for RNA sequencing and quality control. DESeq software was used for differential gene expression (DGE) analysis. qPCR and ELISA verification were performed on DGE analysis.

**Results:**

The DGE profiles of 77 miRNA and 331 mRNA were selected. The GO enrichment analysis comprised 43 biological processes, 49 cell components, and 42 molecular functions. The KEGG enrichment results included 40 KEGG pathways. The PCR results showed that, compared with the Normal group, the miR-4685-3p levels decreased in the CHD_KDBS group (*P* = 0.001), and were found to be lower than those observed in the CHD_NKDBS group. The downstream mRNA C1 regulated by miR-4685-3p showed an increasing trend in the CHD_KDBS group, which was higher than that in the Normal group (*P* = 0.0019). The mRNA C4 and C5 in the CHD_KDBS group showed an upward trend, but the difference was not statistically significant. ELISA was utilized for the detection of proteins associated with the complement and coagulation cascade pathway. It was found that the expression level of C1 was significantly upregulated in the CHD_KDBS group compared with the Normal group (*P* < 0.0001), which was seen to be higher than that in the CHD_NKDBS group (*P* < 0.0001). The expression levels of C4 and C5 in the CHD_KDBS group were significantly lower than the Normal group, and were lower than that in the CHD_NKDBS group (*P* < 0.0001).

**Conclusion:**

The occurrence of CHD_KDBS might be related to the activation of the complement and coagulation cascade pathway, which is demonstrated by the observed decrease in miR-4685-3p and the subsequent upregulation of its downstream C1QC. In addition, the expression levels of complement C4 and C5 were found to be decreased, which provided a research basis for the prevention and treatment of this disease.

## 1. Introduction

The WHO Global Health Estimate Report 2022 (1) highlighted the significant prevalence of cardiovascular-related diseases as a major public health concern. The report indicated that approximately 33.2 million deaths worldwide can be attributed to cancer, cardiovascular disease, diabetes, and chronic respiratory diseases due to the simultaneous increase in population growth and life expectancy. The “Summary of China Cardiovascular Health and Disease Report 2021” (2) also pointed out that the prevalence and mortality rates of cardiovascular diseases in China continue to rise, with rural and urban cardiovascular diseases accounting for 46.74% and 44.26% of the total causes of death, respectively, resulting in an increasing burden on the healthcare system and society as a whole. Unstable angina encompasses a wide range of cardiac conditions, such as NSTEMI, STEMI, and new onset angina. Unstable angina is classified as an acute coronary syndrome, wherein the majority of cases involve partial or total obstruction of the coronary arteries due to the rupture of atherosclerotic plaques and thrombosis, resulting in myocardial ischemia and even myocardial infarction in severe cases (3).

Evidence of kidney deficiency and blood stasis (KDBS) syndrome can occur in patients with vascular dementia (VaD) (4). With the changes in social lifestyle, the traditional Chinese medicine (TCM) syndrome of unstable angina pectoris with coronary heart disease presents a combination form of “kidney deficiency and blood stasis.” A clinical epidemiological survey on the distribution of TCM syndromes showed that a qi deficiency was the main symptom of angina pectoris in coronary heart disease, accounting for up to 78.8%, second only to blood stasis, of which heart and kidney qi deficiency was the primary underlying factor (5). Another study analyzed nine common symptom combinations observed in patients with angina pectoris using algorithms, of which the analysis revealed the symptom group with qi deficiency and blood stasis as the main evidence accounting for as high as 21% (6). However, there is still a lack of screening and validation studies on differential molecules of kidney deficiency and blood stasis associated with coronary heart disease. Previous studies have found that miRNAs exhibit a unique diagnostic value for cardiovascular disease (7–10). The complementarity of miRNAs is paired with the 3’-terminal untranslated regions (3’UTR) of messenger RNA (mRNA) target molecules to form silencing complexes, regulating target gene expression and protein levels (11, 12). A number of studies have analyzed the expression information of differential mRNA in coronary heart disease (13, 14). These molecules play an important role in the pathological stages associated with endothelial injury, inflammatory response, and vascular smooth muscle proliferation of coronary heart disease.

**Figure 1:**
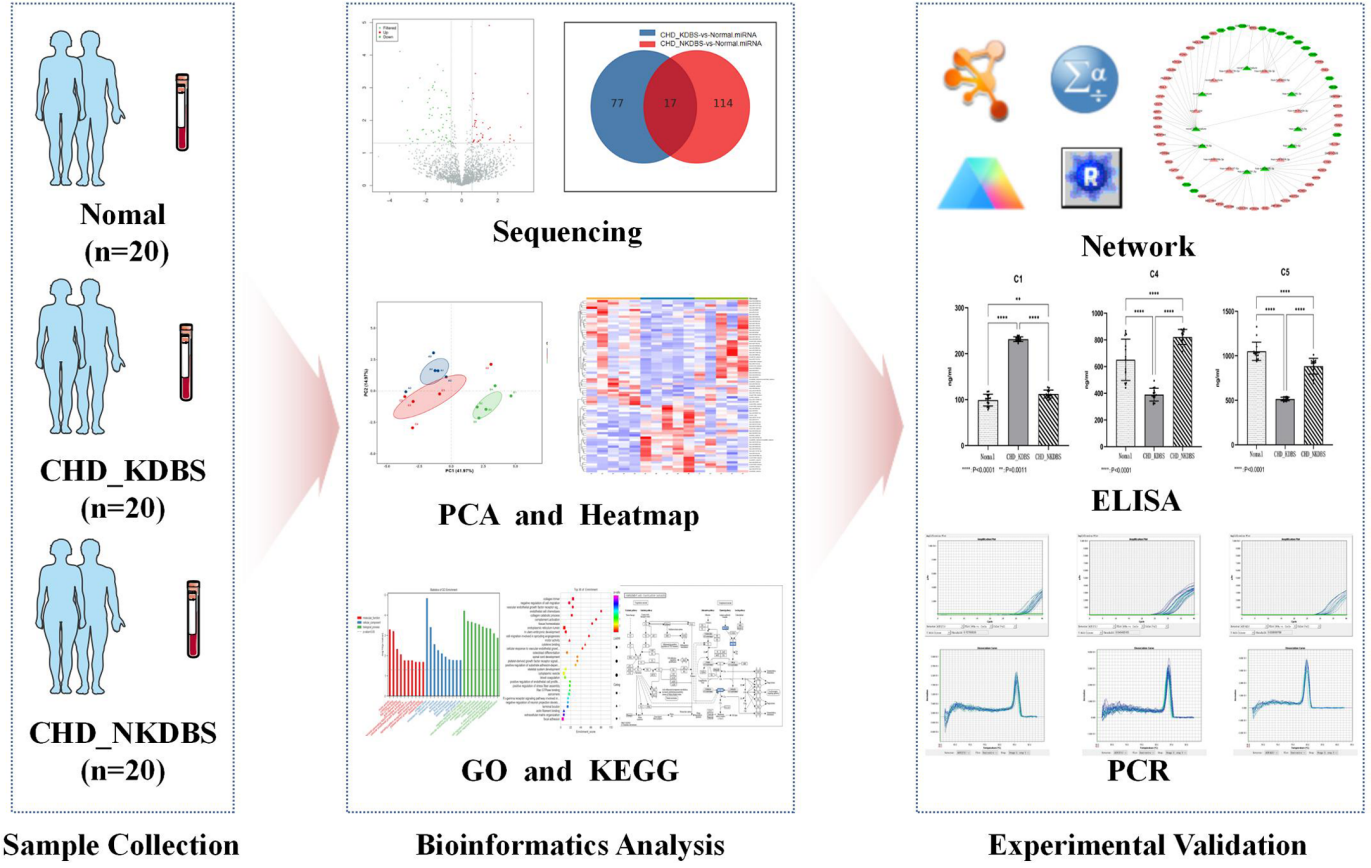
Process overview. In this study, the sample collection was carried out, which included 20 cases of CHD_KDBS participants, 20 cases of CHD_NKDBS participants, and 20 Normal participants. The sequencing of the samples was conducted, and further analysis was performed to examine differential expression using bioinformatics analysis. ELISA and qPCR verification were performed on experimental validation.

The purpose of this study is to screen and establish the miRNA–mRNA differential expression network associated with KDBS syndrome in individuals with unstable angina and coronary heart disease, and to verify the potential biomarkers from protein and transcription levels, thereby offering new basic support for investigating the underlying pathological mechanisms of KDBS syndrome in unstable angina. The flow of the study is shown in Figure 1.

## 2. Materials and methods

### 2.1. Recruitment of participants

The recruited subjects were patients diagnosed with CHD unstable angina pectoris and a control group of healthy subjects. All subjects were treated in the outpatient department and ward of the Cardiovascular Department of Guang'anmen Hospital, Chinese Academy of Chinese Medical Sciences. The recruitment period was from December 2019 to December 2021. Finally, a total of 60 cases were included in the study, comprising 20 cases of CHD_KDBS, 20 cases of CHD_NKDBS, and 20 cases of healthy subjects.

### 2.2. Diagnostic criteria

The diagnostic criteria utilized for patients with unstable angina are based on the “Update on the Guidelines for the management of patients with unstable angina/Non-ST-elevation myocardial infarction” developed by the American College of Cardiology Foundation (ACCF)/American Heart Association (AHA) (15). The evaluation criteria for CHD_KDBS are based on the “Diagnostic Criteria for Symptoms of Angina Pectoris in Coronary Heart Disease” (16) and “Dialectical Diagnostic Criteria for the Main Types of Angina Pectoris in Coronary Heart Disease” (17), which are issued by the Cardiovascular Disease Branch of the Chinese Association of Traditional Chinese Medicine. We also refer to the “Reference Standards for False Evidence Differentiation of Traditional Chinese Medicine” (18) issued by the National Committee for the Study of False Evidence and Geriatrics of Integrated Traditional Chinese and Western Medicine. This clinical trial was approved by the Ethics Committee of Guang'anmen Hospital of the Chinese Academy of Chinese Medical Sciences, and has been registered in the US Clinical Trials Database under registration number ChiCTR2000035358 (https://clinicaltrials.gov/).

### 2.3. Inclusion criteria

The inclusion criteria of the study are as follows: (1) the disease group satisfies the established diagnostic criteria; (2) patients diagnosed with kidney deficiency and blood stasis syndrome according to TCM syndromes (19) (as shown in Supplementary Table S1); (3) patients between the ages of 35 and 85 years old; (4) the healthy control group consists of individuals without liver, kidney, and hematopoietic system diseases, cardiovascular and cerebrovascular diseases, tumors, endocrine diseases, or serious mental diseases; and (5) the participants are required to provide voluntary informed consent prior to participating in the study. Each type of diagnosis must meet at least 1 item from category A and 1 item from category B, and a total score of ≥8 points is required for a diagnosis to be made.

### 2.4. Exclusion criteria

The exclusion criteria of the study are as follows: (1) patients with chest pain that is not caused by coronary heart disease; (2) patients with difficult to control hypertension, severe cardiopulmonary insufficiency, arrhythmias, and malignant tumors; (3) patients with primary diseases of the heart, brain, liver, kidney, and hematopoietic system, as well as patients with insulin-dependent type 2 diabetes mellitus; (4) patients who are prone to allergies, or those who have a documented allergic reaction to a certain component of the test drug; and (5) patients with poor compliance and low possibility of follow-up, or those who have participated in other clinical trials within the past half month.

### 2.5. Sample collection

A volume of 4 ml of venous blood was collected from the participants, and the peripheral blood nuclear cells (PBNCs) were isolated and purified as specimens. Among them, five specimens in each group were used for the high-throughput sequencing of miRNA and mRNA, and the remaining 15 samples were used for enzyme-linked immunosorbent assay (ELISA) and quantitative polymerase chain reaction (qPCR) to verify the key differential genes.

#### 2.5.1. Reagents and instruments

The reagents and instruments employed in this study were as follows: Trizol reagent (Invitrogen Company, 15596018); red cell lysate (TIANGEN Company, RT122-02); FastKing gDNA Dispelling RT SuperMix FastKing (TIANGEN Company, KR118-02); miRcute Plus miRNA First-Strand cDNA Kit miRcute (TIANGEN Company, KR211-02); Ultramicro Spectrophotometer (Thermo Scientific Company, NanoDrop 2000); high speed refrigerated centrifuge (HITACHI Company, 90335001); and adjustable pipette (Eppendorf Company, 0030127722). The Agilent 2100 Bioanalyzer is employed for conducting RNA integrity checks, which is able to quantitatively assess the quality of RNA samples through RNA integrity number (RIN).

#### 2.5.2. Separation and purification of PBNC

The blood samples to be measured were placed into sampling tubes, which were subject to centrifugation at a speed of 3,000 rpm at 4°C for 15 min. The sample was extracted using a pipette gun and retained. Four times the volume of the remaining sample was added into the collection tube, and then it was fully mixed using a disposable plastic straw. It was allowed to stand for 15 min. The samples were divided into 2 ml EP tubes using plastic straws and thereafter subjected to centrifugation at 12,000 rpm at 4°C for 15 min. After centrifugation, the bottom layer of dense parenchyma was nucleated cells, and the top layer was lysated red blood cell (RBC) cytoplasm and RBC lysate. The upper layer of the EP tube fluid was carefully extracted using a pipette gun and discarded, while ensuring minimal contamination of the RBC cytoplasm without being absorbed into the bottom nucleated cells. A red blood cell lysate was added to 1.5 ml, mixed thoroughly using plastic straws, allowed to stand for 15 min, and then centrifuged at 12,000 rpm at 4°C for 15 min. The process was repeated until the supernatant in the EP tube became clear and the underlying cells were basically free of red blood cells. A pipette gun was used to absorb the supernatant ,which was subsequently discarded. A 1 ml Trizol was added and mixed fully with the remaining nucleated cells. The resulting mixture was then stored and frozen in a refrigerator at −80°C.

### 2.6. High-throughput sequencing

RNA was extracted using the mirVana miRNA Isolation Kit (Ambion) in accordance with the manufacturer's guidelines. RNA integrity was evaluated using the Agilent 2100 Bioanalyzer (Agilent Technologies, Santa Clara, CA, USA). The samples with an RIN of ≥7 were subjected to the subsequent analysis. The libraries were constructed using TruSeq Stranded Total RNA with Ribo-Zero Gold according to the manufacturer's instructions. Subsequently, these libraries were subjected to sequencing using the Illumina sequencing platform (HiSeqTM 2500 or other platform), resulting in the generation of 150 bp/125 bp paired-end reads.

#### 2.6.1. RNA extraction

The lysate was treated with 600 μl of Lysis/Binding Buffer. The homogenate was treated with 30 μl of miRNA Homogenate Additive and mixed well by vortexing or inverting the tube several times. The mixture was left on ice for 10 min. A volume of acid phenol–chloroform, equal to the lysate volume before the addition of the miRNA Homogenate Additive was added. The mixture was centrifuged for 5 min at a maximum speed (10,000 × *g*) to separate the aqueous and organic phases. The aqueous (upper) phase was carefully extracted from the solution without disturbing the lower phase, thereafter transferred to a new tube; 1.25 volumes of room temperature 100% ethanol (EtOH) were then added to the aqueous phase. The lysate/ethanol mixture from the previous step was pipetted onto the filter cartridge. Centrifugation was done for 30 s at 13,000 rpm to pass the mixture through the filter. The flow-through was discarded. A 350 μl of miRNA Wash Solution 1 was applied to the filter cartridge and centrifuged for 30 s at 13,000 rpm. The flow-through from the collection tube was discarded, and the filter cartridge was replaced into the same collection tube. The 10 μl of DNase I and 70 μl of Buffer RDD QIAGEN (#79254) were mixed. The mixture was added to the filter cartridge and left at room temperature for 15 min. After that, 350 μl of miRNA Wash Solution 1 was applied to the filter cartridge and centrifuged for 30 s at 13,000 rpm. The flow-through from the collection tube was discarded, and the filter cartridge was replaced into the same collection tube. A Wash Solution 2/3 (500 μl) was applied to the filter cartridge and centrifuged for 30 s at 13,000 rpm. The assembly was spun for 1 min to remove the residual fluid from the filter. The filter cartridge was then transferred into a fresh collection tube. A pre-heated (95°C) elution solution (100 μl) was applied to the center of the filter. It was left at room temperature for 2 min and then spun for 20–30 s at a maximum speed to recover the RNA. The eluate, which contains the RNA, was collected and stored at −70°C.

#### 2.6.2. Ribo-Zero deplete and fragment RNA

Each well with 10 μl total RNA (1 μg) was added with 5 μl of rRNA Binding Buffer and 5 μl of rRNA Removal Mix-Gold. The entire volume was gently pipetted up and down six times to mix the solution thoroughly. The PCR plate was then incubated at 68°C for 5 min followed by placing it at room temperature for 1 min. Next, 35 μl of rRNA Removal Beads was added to each well of a new PCR plate. Using a pipette adjusted to 45 μl, the solution was pipetted quickly up and down 20 times with the tip of the pipette placed at the bottom of the well to ensure thorough mixing. This was followed by incubating at room temperature for 1 min. The PCR plate was placed on a magnetic stand at room temperature for 1 min. The supernatant from each well was transferred to the corresponding well of the new PCR plate. Then, 99 μl of RNAClean XP beads was added to each well of the PCR plate. The entire volume was gently pipetted up and down 10 times to mix the solution thoroughly. The PCR plate was incubated at room temperature for 15 min and then placed on the magnetic stand for 5 min. All of the supernatant from each well of the PCR plate was removed and discarded. A 200 μl of freshly prepared 70% EtOH was added to each well, followed by incubating the PCR plate at room temperature for 30 s. The supernatant was then removed and discarded from each well. The PCR plate was allowed to stand at room temperature for 15 min to dry. To each well of the PCR plate, 11 μl of Elution Buffer was added. The entire volume was gently pipetted up and down 10 times to mix the solution thoroughly. The PCR plate was incubated at room temperature for 2 min, and then placed on the magnetic stand for 5 min. From the PCR plate, 8.5 μl of supernatant was transferred to a new 0.2 ml tube. To each well of the plate, 8.5 μl of Elute, Prime, Fragment High Mix was added. The entire volume was gently pipetted up and down six times to mix the solution thoroughly. The plate was incubated at 94°C for 8 min, held at 4°C, and then centrifuged briefly.

#### 2.6.3. Synthesize first-strand cDNA

In each well of the PCR plate, 8 μl of First-Strand Synthesis Act D Mix and SuperScript II Reverse Transcriptase were added. The entire volume was gently pipetted up and down six times to mix the solution thoroughly. The mixture was then incubated at the following temperatures: 25°C for 10 min, 42°C for 15 min, 70°C for 15 min, and hold at 4°C.

#### 2.6.4. Synthesize second strand cDNA

In each well of the PCR plate, 5 μl of End Repair Control (2 μl End Repair Control + 98 μl Resuspension Buffer) was added, and 20 μl of Second Strand Marking Master Mix was added. The entire volume was gently pipetted up and down six times to mix the solution thoroughly. It was incubated at 16°C for 60 min. The plate was removed from the thermal cycler, and 90 μl of mixed AMPure XP beads was added to each well of the plate. The entire volume was gently pipetted up and down 10 times to mix the solution thoroughly. The plate was incubated at room temperature for 15 min. It was placed on the magnetic stand at room temperature for 5 min. Then, 135 μl of supernatant from each well of the plate was removed and discarded. With the plate on the magnetic stand, 200 μl of freshly prepared 80% EtOH was added to each well. The plate was incubated at room temperature for 30 s, and then all of the supernatant from each well was removed and discarded. The plate was let to stand at room temperature for 15 min to dry, and then the plate was removed from the magnetic stand. Subsequently, 17.5 μl of Resuspension Buffer was added to each well of the plate. The entire volume was gently pipetted up and down 10 times to mix the solution thoroughly. The plate was incubated at room temperature for 2 min. Then, the plate was placed on the magnetic stand at room temperature for 5 min, and 15 μl of supernatant from the plate was transferred to the new PCR plate.

#### 2.6.5. Adenylate 3’ ends

A 2.5 μl of diluted A-Tailing Control (1 μl A-Tailing Control + 99 μl Resuspension Buffer) was added to each well of the plate, and a 12.5 μl of A-Tailing Mix was added to each well of the plate. The entire volume was gently pipetted up and down 10 times to mix the solution thoroughly. The mixture was incubated according to the following progress: 37°C for 30 min, 70°C for 5 min, and then held at 4°C.

#### 2.6.6. Ligate adapters

A 2.5 μl of diluted Ligation Control (1 μl Ligation Control + 99 μl Resuspension Buffer) was added to each well of the plate, and a 2.5 μl of Ligation Mix was added to each well of the plate. Furthermore, a 2.5 μl of RNA Adapter Index was added to each well of the plate. The entire volume was gently pipetted up and down 10 times to mix the solution thoroughly. The sample was incubated at 30°C for 10 min, and then a 5 μl of Stop Ligation Buffer to was added each well of the plate. The entire volume was gently pipetted up and down 10 times to mix the solution thoroughly.

#### 2.6.7. Enrich DNA fragments

A 5 μl of PCR Primer Cocktail was added to each well of the PCR plate, followed by the addition of a 25 μl of PCR Master Mix to each well of the PCR plate. Gently pipette the entire volume up and down to mix the solution thoroughly. The mixture was incubated in the following progress: one cycle at 98°C for 30 s; 15 cycles at 98°C for 10 s, 60°C for 30 s, and 72°C for 30 s; one cycle at 72°C for 5 min; and then hold at 10°C. A sample volume of 1 μl was loaded on an Agilent Technologies 2100 Bioanalyzer. The size and purity of the sample were checked.

#### 2.6.8. Quality test

RNA purity and concentration were determined using NanoDrop 2000, which was zeroed with DEPC water in advance, and then measured. A total RNA sample of 2.5 μl was taken, 650 μl DEPC water was added, and the mixture was measured for optical density (OD) values at 260 nm and 280 nm. The determination and generation of the result file were automatically completed for the tested sample. The sample RNA concentration (μg/ml) was calculated using the formula: RNA (μg/ml) = OD260 × sample dilution × 40. The purity of RNA could be estimated based on the OD260/OD280 ratio, which is approximately 2.0 for pure RNA. A low ratio indicated the presence of residual proteins, while a high ratio indicated RNA degradation.

### 2.7. qPCR validation

The samples with Trizol were stood at room temperature for 10 min and were centrifuged at 12,000 rpm/min at 4°C for 10 min. After the precipitate was discarded, the supernatant was aspirated into a new tube. After pre-cooling the chloroform in advance, 100 μl was added to the new tube, vigorously mixed, and it stood at room temperature for 5 min. The sample was centrifuged at 12,000 rpm/min at 4°C for 15 min, and the supernatant was transferred to a new tube, mixed well, and then the mixture was stored at −20°C overnight. The sample was then taken out and centrifuged at 12,000 rpm/min at 4°C for 15 min, and the supernatant was discarded. An 800 μl of pre-cooled 75% ethanol was added and mixed well, centrifuged at 12,000 rpm/min at 4°C for 10 min, and the supernatant was discarded. After discarding the supernatant for the last time, it was dried for 30 min; 30 μl of DEPC water was then added, and it was solubilized in water at 55°C for 10 min. After mixing, 6 μl of the sample was taken and added to 1.5 ml of distilled water, mixed well, and the OD value (260 nm, 280 nm) was measured. The remaining samples were stored at −20°C or immediately reverse-transcribed.

The primer sequences used for the human target genes were as follows: hsa-miR-4685-3p sequences (UCUCCCUUCCUGCCCUGGCUAG), C1QC Forward (GAGGGCAGATACAAGCAGAA), C1QC reverse (GGGACTTTGCAGGTGAACTT), C4B Forward (TCCAGGTGCCCTTGAAAGAT), C4B reverse (AGGGTTGTAAATGGGCTGGT), C5 Forward (CTGTCAAGGCAAAGGTGTTCA), C5 reverse (ACATTTGGAGGACTTTGTGCC), β-actin Forward (GAGACCTTCAACACCCCAGCC), and β-actin reverse (AATGTCACGCACGATTTCCC).

### 2.8. ELISA testing

Additional evidence is provided by using ELISA to complement the results of differential gene expression (DGE) analysis. Specifically, we hope that this approach can be used to assess whether changes in gene expression observed at the RNA level correspond to changes at the protein level, as shown in the ELISA results. We hope this can serve as a validation step to enhance the robustness of the DEG results.

#### 2.8.1. Reagent and sample pretreatment and preparation

The sample extracted from the protein was serum, which was isolated using a sterile test tube without coking or endotoxin during the procedure. The blood was collected by venipuncture and rapidly centrifuged (3,000 × *g* centrifugation for 10 min). The platform we used for ELISA was Elabscience. Each component reagent and the sample to be tested were equilibrated at room temperature. The concentrated cleaning solution was diluted with deionized water according to the dilution ratio of 1:10 for reserve. The number of required micropores was determined, and marks were made.

#### 2.8.2. Procedure of testing

The treated samples were divided into 40 μl samples in sterile tubes. After labeling, 10 μl of sample balancing solution was added to each sample to be tested. The samples were mixed at room temperature and balanced for 30 min. The micropores were successively added with 50 μl standard substances of each concentration. The treated samples were successively added with 50 μl into each hole. The microplate was covered with a sealing plate film, incubated smoothly in a 37°C incubator for 60 min, and then the residual liquid in the holes was fully discarded and rinsed five times with the pre-diluted cleaning solution. Each hole was successively added with 50 μl of color developer I and then mixed thoroughly by adding 50 μl of color developer II. The reaction took place at room temperature for 15 min under dark conditions. After the reaction, 50 μl of termination solution was added to each hole. After the experiment was terminated, the absorbance value of each hole was read at the band of 450 nm with the enzyme label instrument within 10 min. The OD value was recorded, and its corresponding concentration was screened out.

### 2.9. Bioinformatics and statistical analysis

After obtaining the differentially expressed miRNA and mRNA of each group, the interaction network was constructed by combining the regulatory relationship between each gene. The starBase v2.0 target gene database (http://starbase.sysu.edu.cn/) was used to optimize the matching and cross-validation of the sequences between miRNA and mRNA, and the reliable potential target genes were screened out and combined into a gene regulatory network. FastQC software was used for quality control check of sequencing results. For statistical analysis, DESeq software was used for differential gene expression analysis, and finally the differential genes were screened according to variance multiplier. FDR error was used for control method to test correction for *P*-value. Due to fewer target genes screened out after two times difference, the conditions for difference was set as *P* < 0.05, and the difference multiple was greater than 1.5 (|log2FC| > 0.58 and *P* < 0.05).

The differential gene screening, cluster analysis, and principal component analysis (PCA) were performed to visualize the data. The analysis tools GO (http://www.geneontology.org/), KEGG (http://www.kegg.jp/), and DAVID (https://david.ncifcrf.gov/) were employed for functional enrichment and signal pathway enrichment analysis. In qPCR studies, the relative expression of genes was calculated using the 2^−ΔΔCt^ calculation method: Relative expression = 2^−ΔΔCt^; ΔΔCt = (Ct target gene-Ct housekeeper gene) experimental group-(Ct target gene-Ct housekeeper gene) control group. SPSS 24.0 statistical software was used for statistical analysis of the remaining experimental data, and the measurement data were expressed as mean ± standard deviation.

## 3. Results

### 3.1. Baseline characteristics of clinical data

The clinical data of 60 individuals were statistically analyzed in this study. Compared with the Normal group, the CHD_KDBS group had decreased high-density lipoprotein (HDL), low-density lipoprotein (LDL), creatinine (CR), and D-dimer. In addition, there were significant differences (*P* < 0.05) observed, indicating that these patients may have high blood lipids, decreased kidney function, and hypercoagulation.

There were significant differences observed in HDL and D-dimer (*P* < 0.05) between the CHD_KDBS and CHD_NKDBS group. The activated partial thromboplastin time (APTT) and prothrombin time (PT) was observed to decrease in the CHD_KDBS group, but there was no significant difference observed with the Normal group, as shown in Supplementary Table S2.

### 3.2. Quality assessment of total RNA and sequencing data

The total RNA extracted from 15 high-throughput sequencing specimens (CHD_KDBS group, CHD_NKDBS group, Normal group, with five cases from each group) were detected by UV absorption method and agarose gel electrophoresis. The results showed that OD260/280 of total RNA in all samples ranged from 1.8 to 2.0. This indicated that the protein or other organic matter contamination degree in RNA samples was low, which met the quality requirements of RNA high-throughput sequencing. In our experiments, it was observed that all RNA samples had RIN values above 6.0, which is generally considered to meet the sequencing requirements (Supplementary Table S3).

After sequencing, the low quality areas of RawReads of the original sequence were removed, and the desired high-quality data CleanReads were obtained after filtration, with the quantity between 91.20 and 101.03 M and the CleanBases between 12.83 and 14.60 G. Q30 represents the number and proportion of bases with a single base error rate of 0.001. After quality control, the Clean Q30 is controlled above 85% to ensure the reliability of sequencing results, as shown in Supplementary Table S4.

### 3.3. Differential miRNA–mRNA in coronary heart disease with kidney deficiency and blood stasis syndrome

According to the screening criteria of differential miRNA and mRNA (|log2FC| > 0.58 and *P* < 0.05), the high-quality sequencing data were statistically analyzed, and finally the miRNA and mRNA were obtained with differences among the test groups.

#### 3.3.1. CHD_KDBS vs. Normal

**Figure 2:**
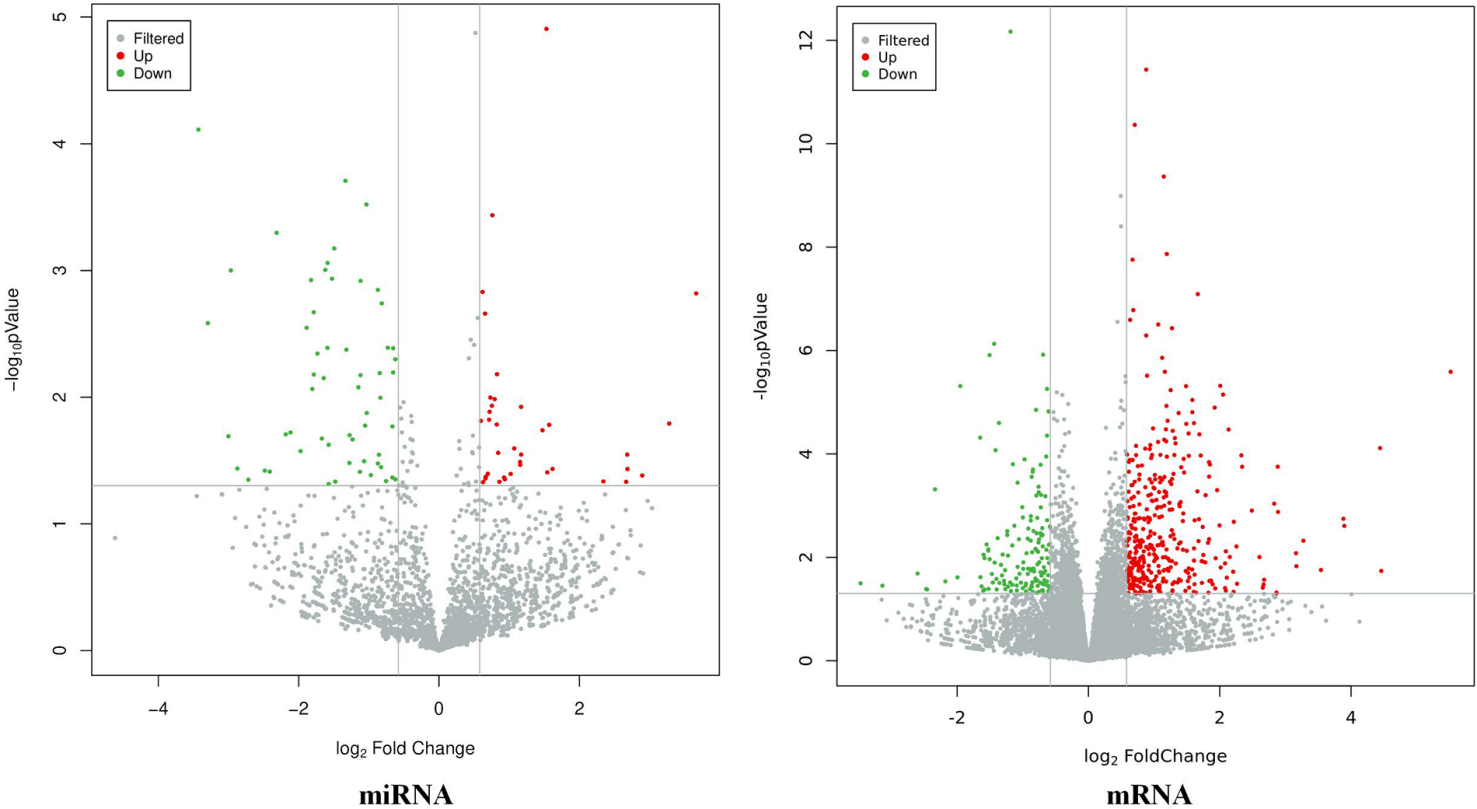
Volcano map of differential genes (CHD_KDBS vs. Normal, *P*-value < 0.05 and |log_2_FC| > 0.58).

The results showed that there were a total of 94 differentially expressed miRNAs (37 upregulated and 57 downregulated) and 537 differentially expressed mRNAs (385 upregulated and 152 downregulated) between the CHD_KDBS group and the Normal group, as shown in Figure 2.

#### 3.3.2. CHD_NKDBS vs. Normal

**Figure 3:**
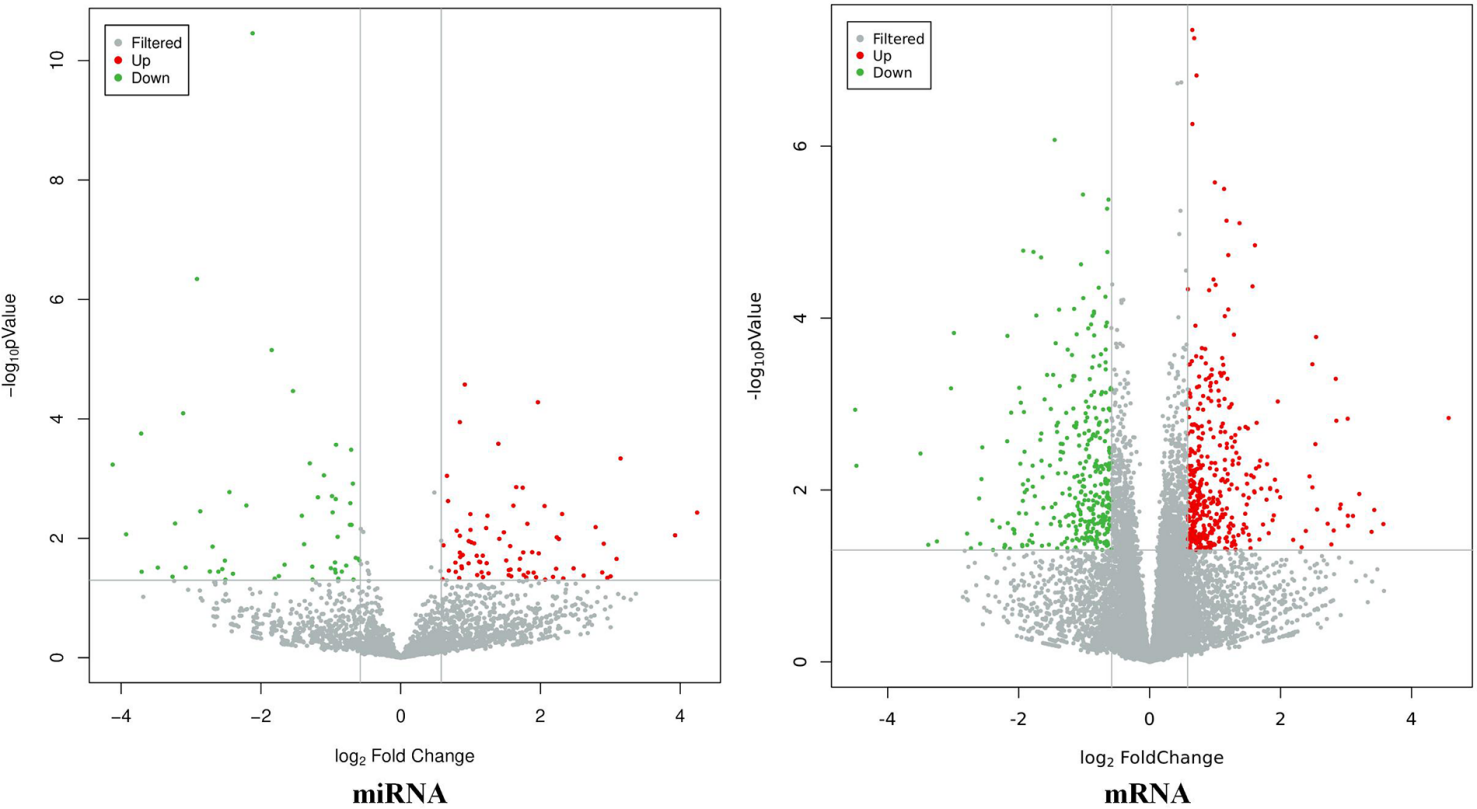
Volcano map of differential genes (CHD_NKDBS vs. Normal, *P*-value < 0.05 and |log_2_FC| > 0.58).

The results showed that there were a total of 131 differentially expressed miRNAs (77 upregulated and 54 downregulated) and 746 differentially expressed mRNAs (399 upregulated and 347 downregulated) between the CHD_KDBS group and the Normal group, as shown in Figure 3.

#### 3.3.3. Differential gene screening for CHD_KDBS

Genes that differ between the CHD_KDBS and Normal group include the following: (1) specific genes related to CHD_KDBS; (2) specific genes associated with CHD; (3) specific genes related to kidney deficiency and blood stasis syndrome; and (4) other genes. The differential genes between the CHD_NKDBS and Normal group included the following: (1) specific genes related to CHD_NKDBS; (2) specific genes associated with CHD; (3) specific genes related to non-kidney deficiency and blood stasis syndrome; and (4) other genes.

**Figure 4:**
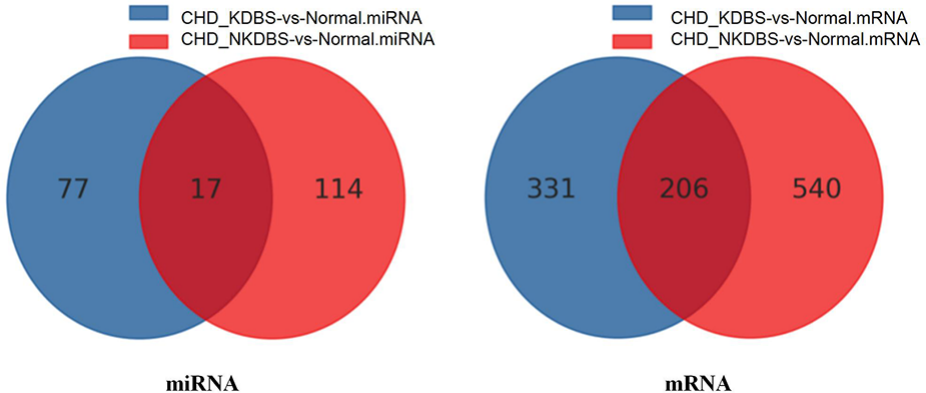
Venn graph of differential genes.

When comparing the two disease groups, the results are KDBS-specific genes. These genes have the potential to distinguish CHD-related genes. Our aim is to analyze the specific genes associated with coronary heart disease in the KDBS group by comparing the two disease groups and the healthy group. The gene obtained by this formula is the final specific difference gene of CHD_KDBS: CHD_KDBS vs. Normal (CHD_KDBS vs. Normal ∩ CHD_NKDBS vs. Normal) (Figure 4). Finally, the differential genes associated with CHD_KDBS included 77 miRNAs (30 upregulated, 47 downregulated) and 331 mRNAs (239 upregulated, 92 downregulated).

#### 3.3.4. Hierarchical clustering and principal component analysis

**Figure 5:**
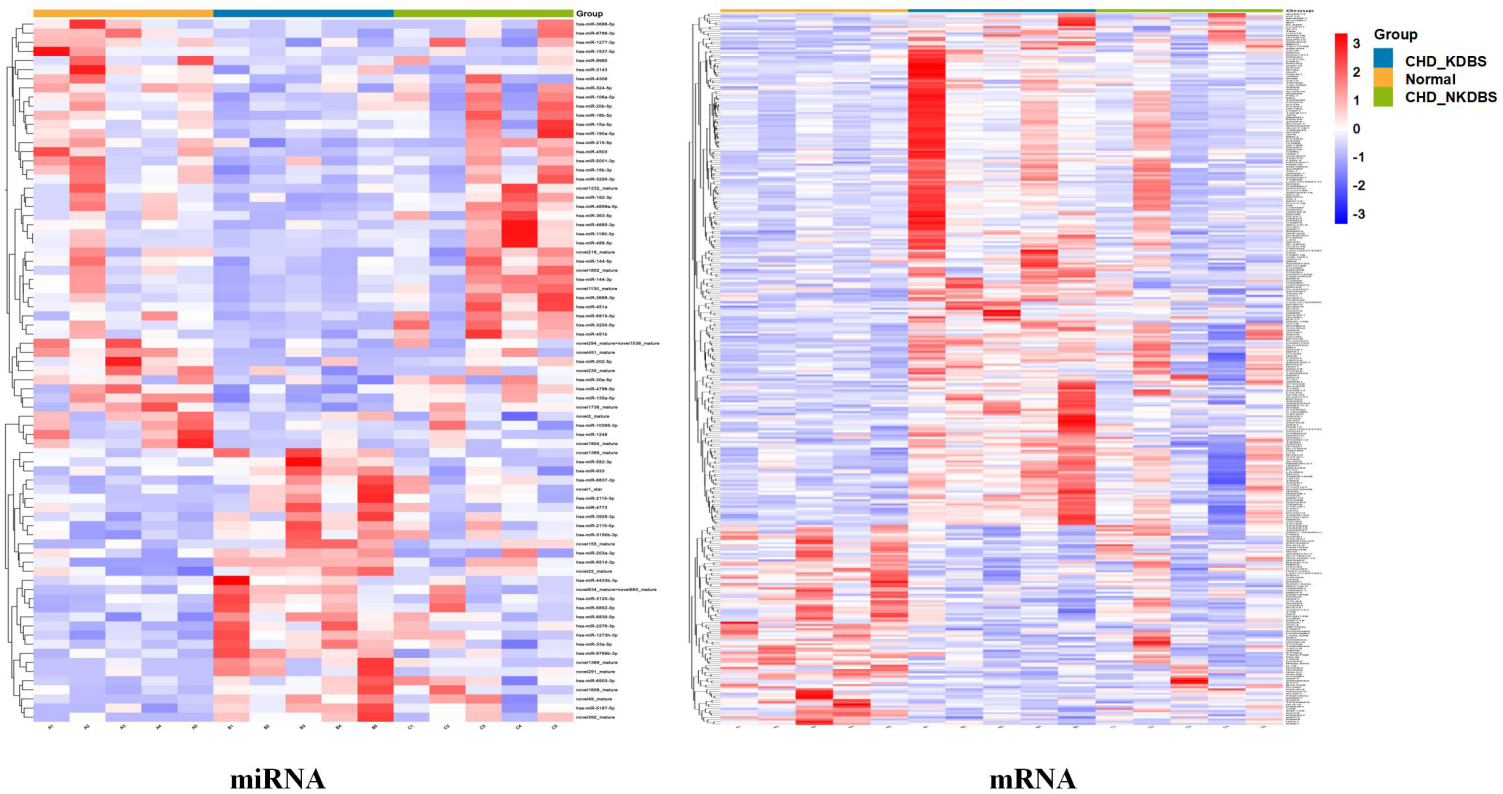
Cluster heatmap of differential genes (CHD_KDBS, CHD_NKDBS, and Normal group).

**Figure 6:**
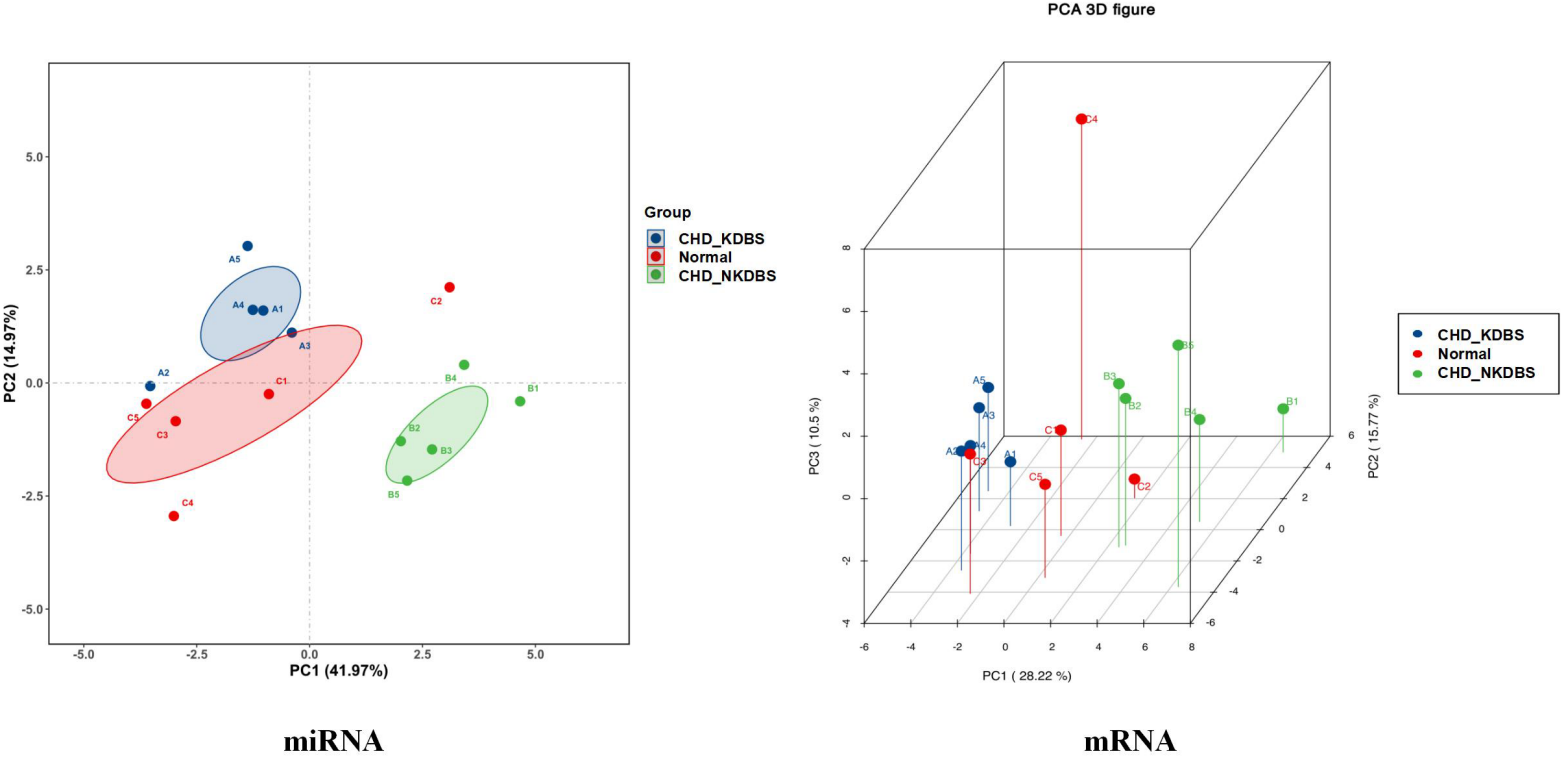
PCA results of differential genes (CHD_KDBS, CHD_NKDBS, and Normal group).

The differential miRNA and mRNA were subjected to stratified clustering analysis and principle component analysis in order to compare the CHD_KDBS, CHD_NKDBS, and Normal groups. The heat map is shown in Figure 5. The red part represents the high-expression gene, and the blue part represents the low-expression gene. The treemap at the top of the picture represents the clustering results of different samples from three different experimental subgroups (CHD_KDBS, Normal, and CHD_NKDBS), and the treemap on the left represents the clustering results of different genes from different samples. The CHD_KDBS group compared with the Normal group showed obvious differences in the comparison of miRNA and mRNA. The PCA results were similar to the cluster analysis results, and the three groups of samples demonstrated good discrimination (Figure 6).

#### 3.3.5. Construction of miRNA–mRNA regulatory network

**Figure 7:**
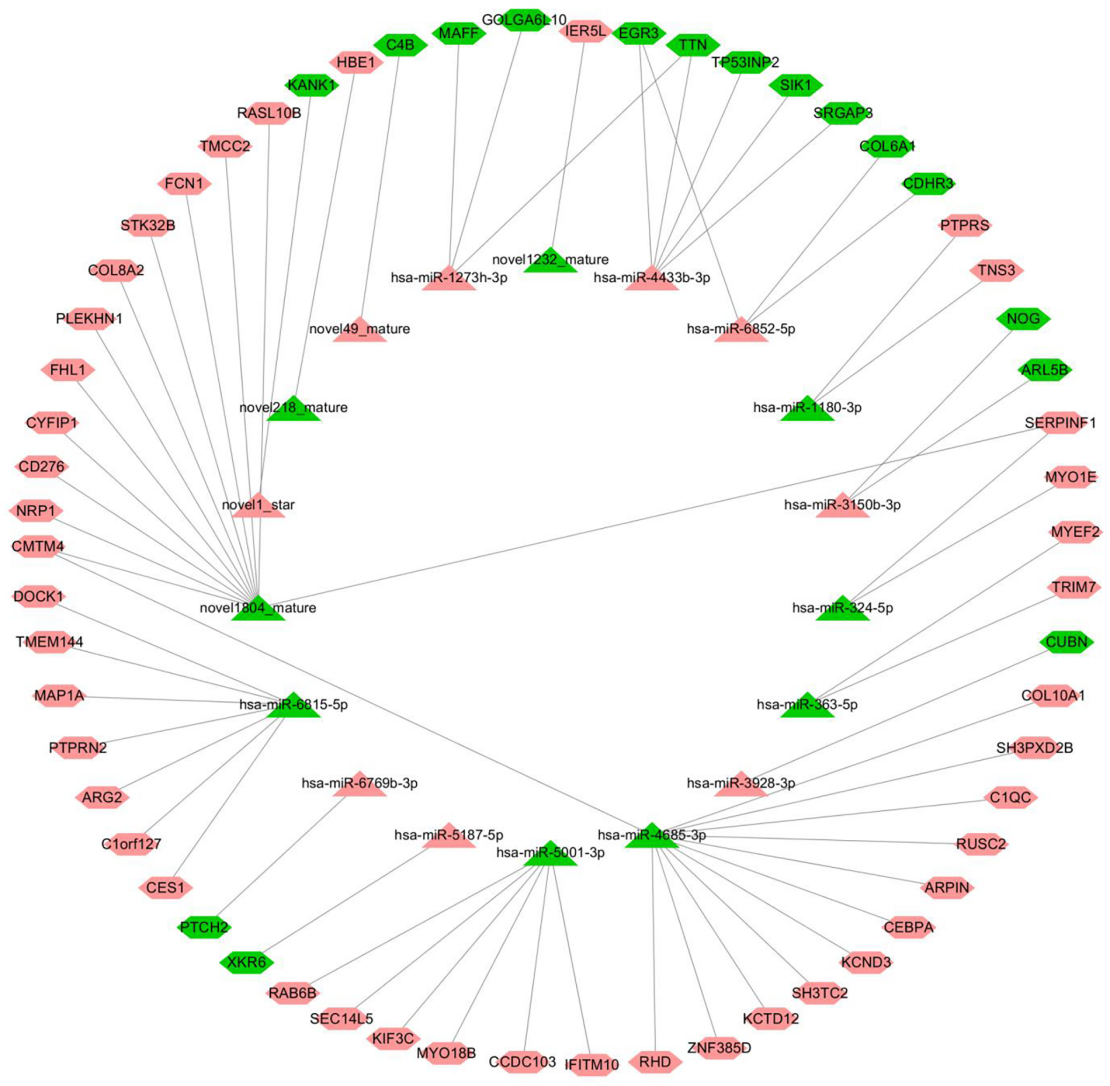
miRNA–mRNA regulatory network (triangles represent miRNAs, hexagons represent mRNAs, the red color indicates upregulated, and the color green indicates downregulated).

The starBase v2.0 target gene database (http://starbase.sysu.edu.cn/) was used to predict and build a network of miRNA–mRNA interactions. According to the intermolecular regulatory conditions, a total of 18 miRNAs (nine downregulated, nine upregulated) and 59 mRNAs (16 downregulated, 43 upregulated) were screened, covering 63 intermolecular regulatory relationships after combination (Figure 7).

The node information of the miRNA–mRNA co-regulatory network is as follows, in which hsa-miR-4685-3p is downregulated and the corresponding C1QC mRNA is upregulated (Supplementary Table S5).

#### 3.3.6. Analysis of GO and KEGG enrichment functions of differential genes

**Figure 8:**
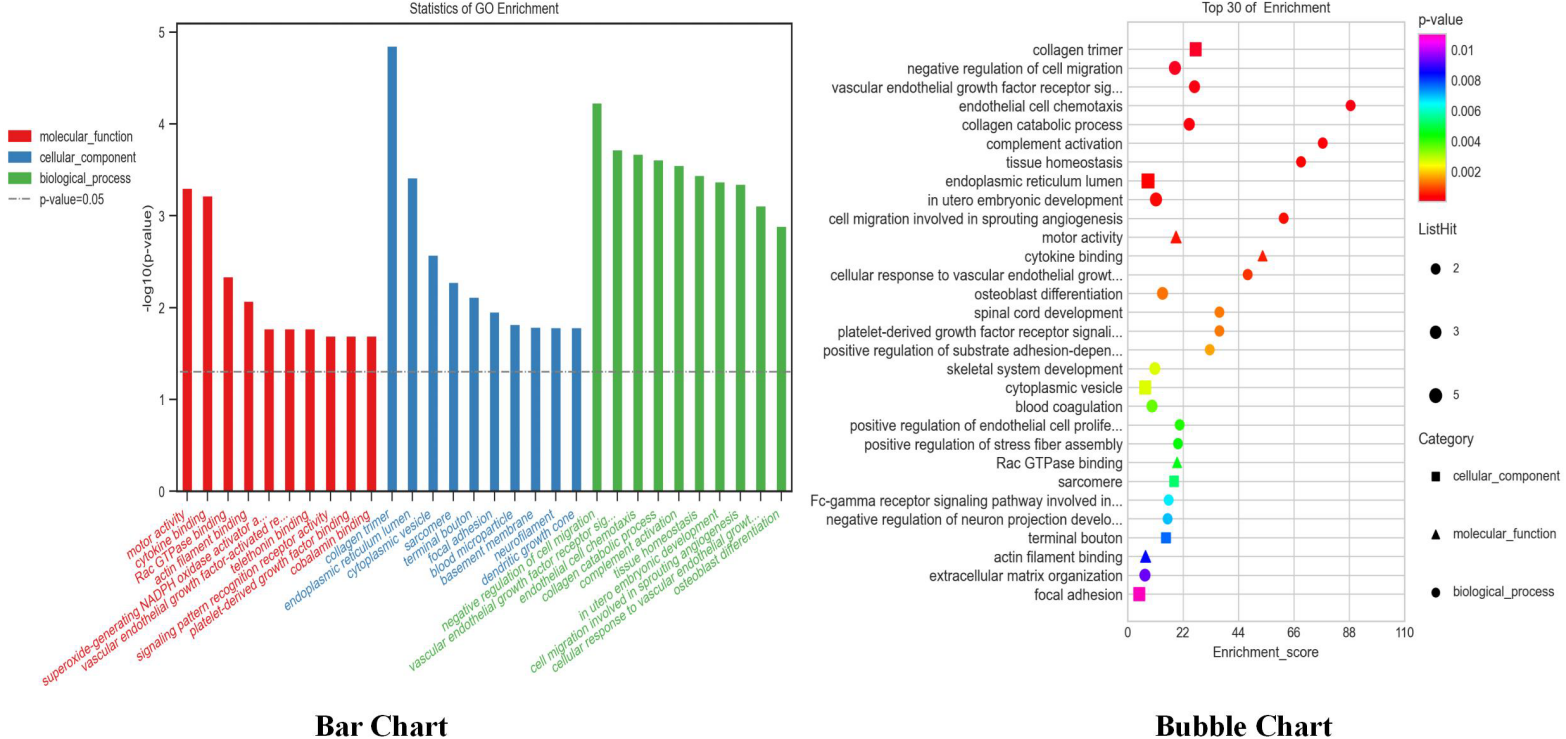
GO enrichment of mRNA (top 10 *P*-values in each category).

**Figure 9:**
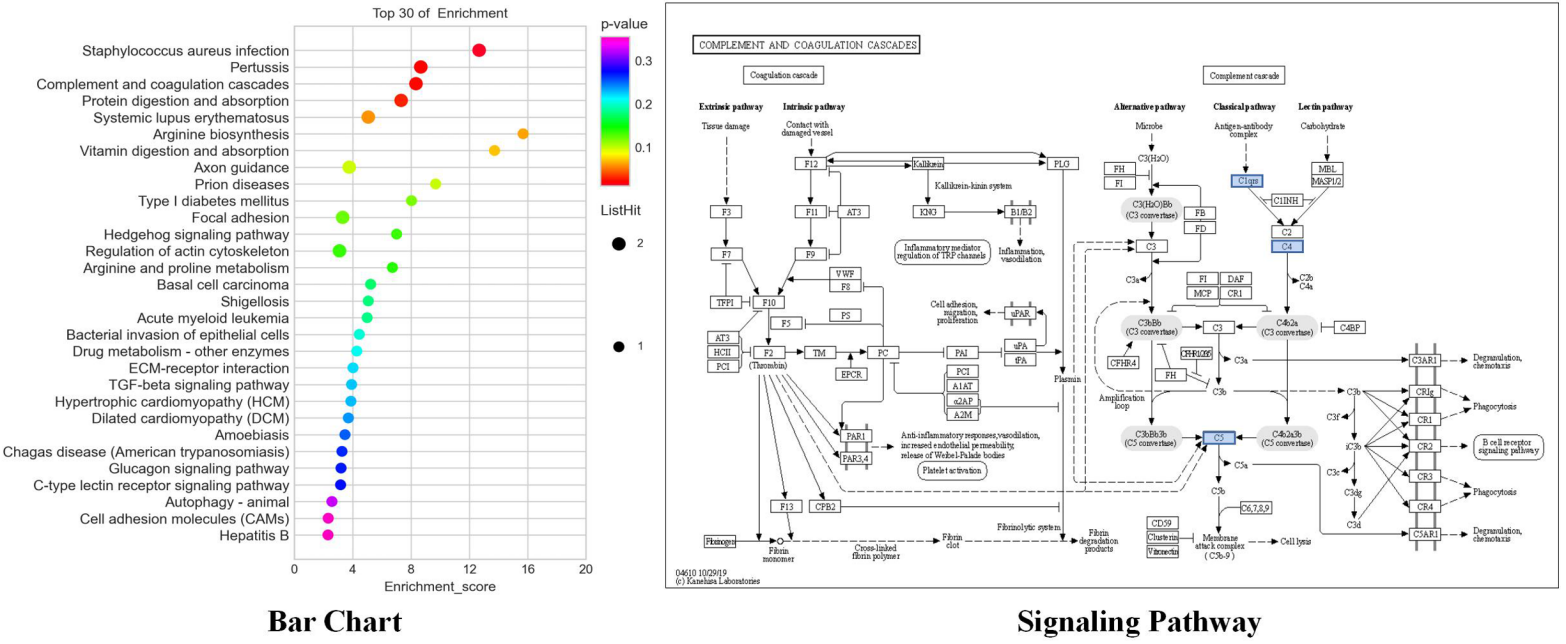
KEGG enrichment of mRNA (top 10 *P*-values in each category).

GO enrichment was used to analyze the differentially expressed mRNA, including 43 biological processes, 49 cellular components, and 42 molecular functions that were significantly expressed in apoptotic cell recognition, negative regulation of cell migration, signal pattern recognition receptor activity, etc. (Figure 8). KEGG enrichment was used to analyze the differentially expressed mRNA, and the results showed that there were a total of 40 KEGG pathways. The top 10 pathways were according to the *P*-value ranking, as shown in Figure 9. The complement and coagulation cascades pathways (path: hsa04610) were selected for experimental verification based on our previous research basis (20).

#### 3.3.7. Verification of qPCR

**Figure 10:**
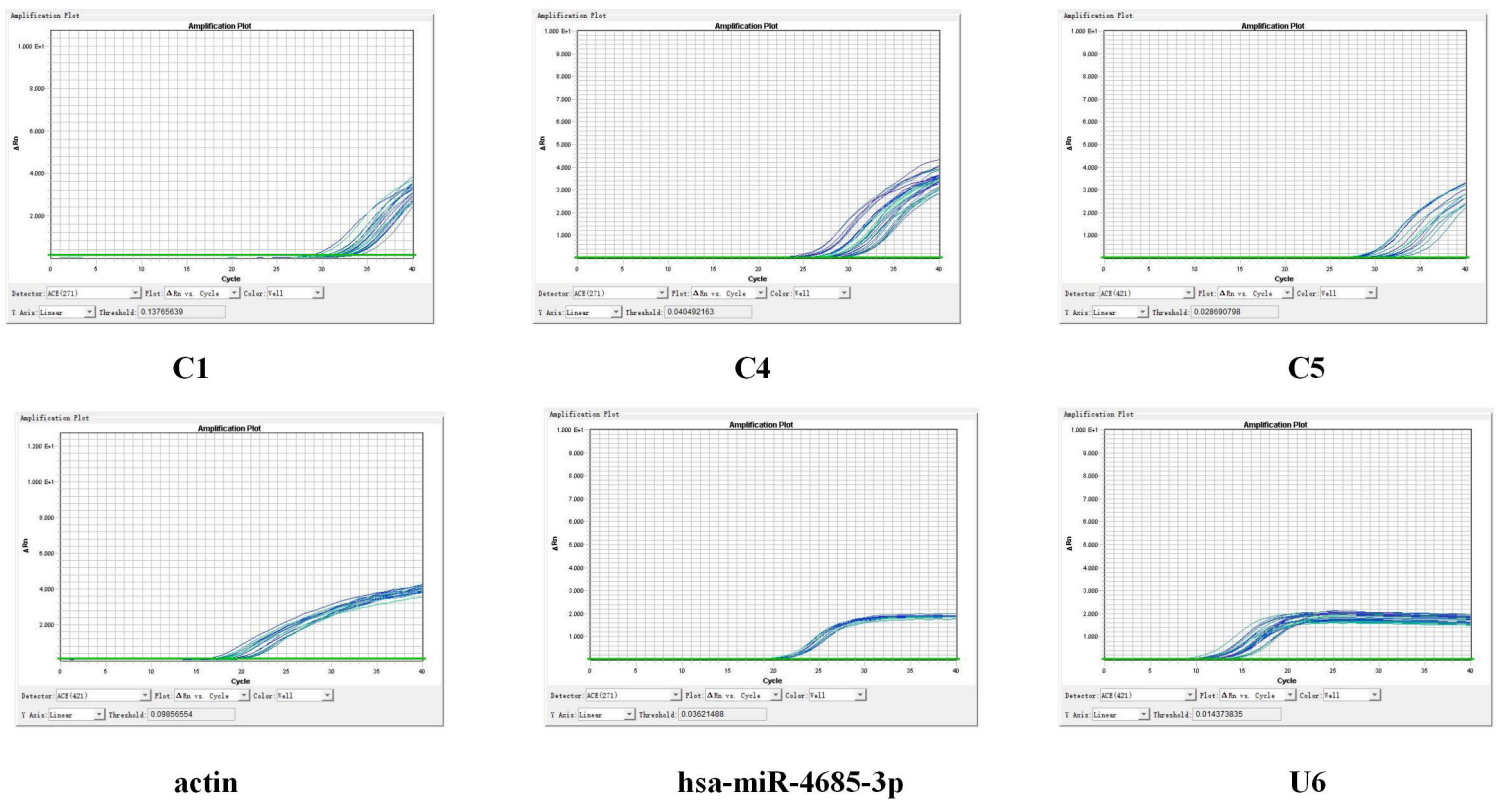
Amplification curve.

**Figure 11:**
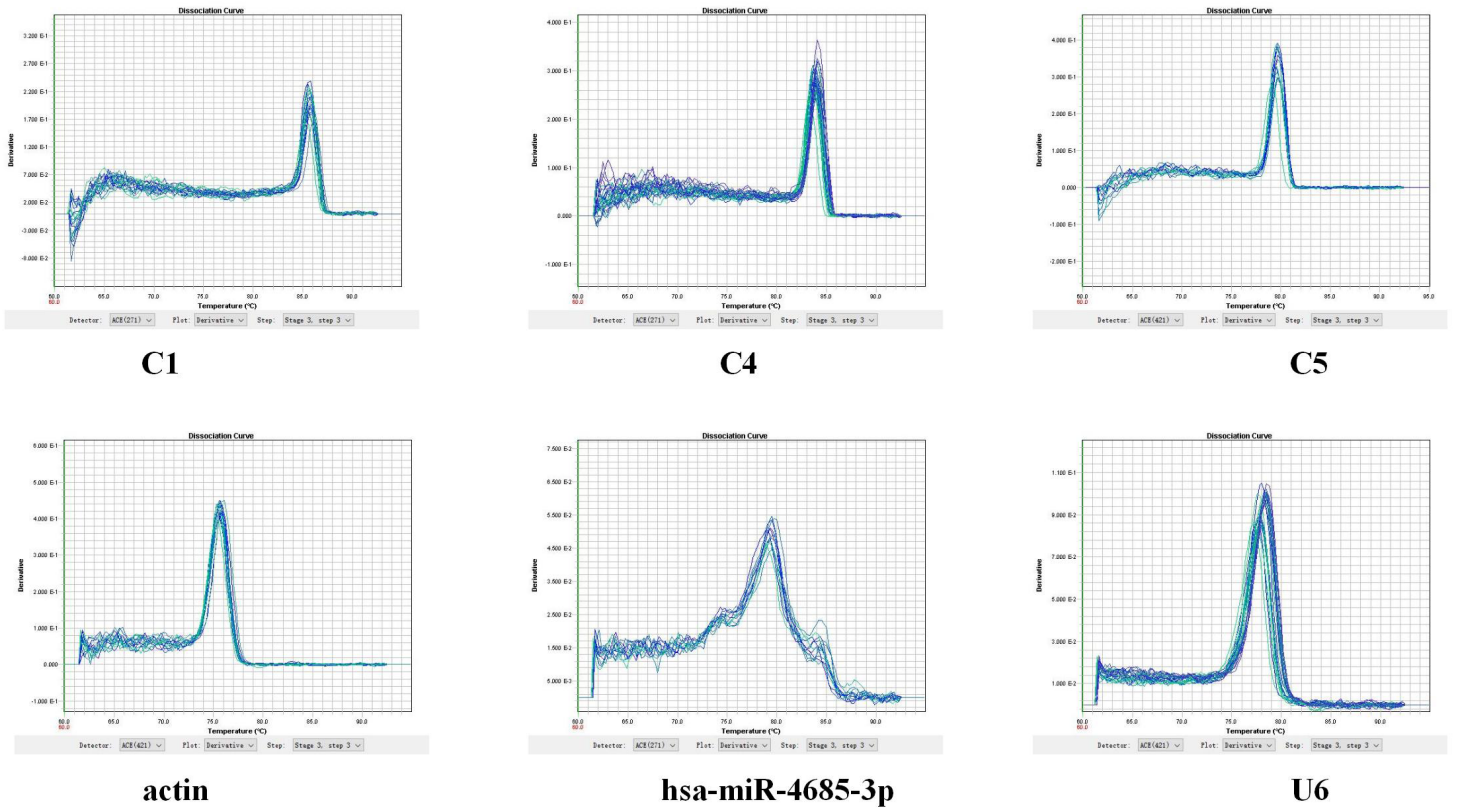
Melting curve.

**Figure 12:**
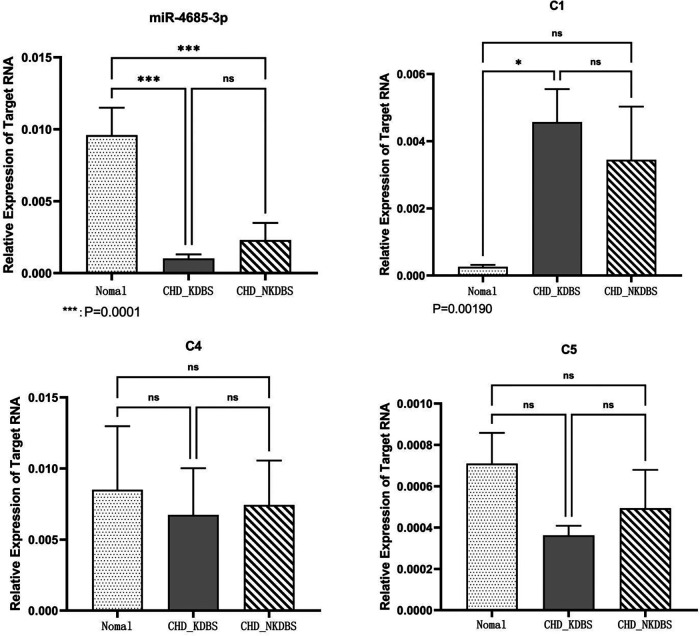
Transcription expression level.

qPCR was used to detect the expression of key molecules (Figures 10–12, Supplementary Table S6). Compared with the Normal group, miR-4685-3p showed a downward trend in the CHD_KDBS group (*P* = 0.001), and was lower than that in the CHD_NKDBS group. The downstream mRNA C1 regulated by miR-4685-3p showed an increasing trend in the CHD_KDBS group, which was higher than that in the Normal group (*P* = 0.0019). The mRNA C4 in the CHD_KDBS group showed an upward trend, but the observed difference was not statistically significant. The mRNA C5 exhibited a downward trend in the CHD_KDBS group, and was comparatively lower than that observed in the other two groups. However, these differences did not reach statistical significance.

#### 3.3.8. Verification of ELISA

**Figure 13:**
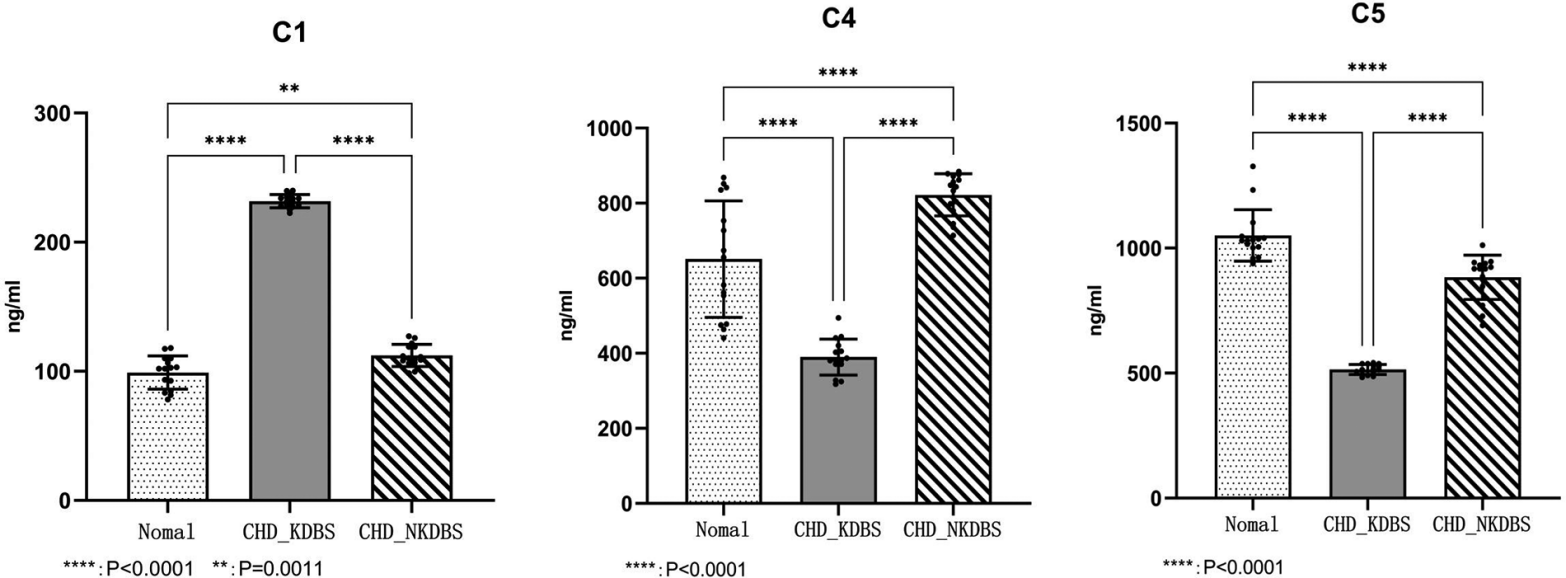
Protein expression level.

The detection of proteins associated with the complement and coagulation cascade pathway was observed using ELISA testing, in accordance with the results from previous sequencing and qPCR analyses. It was found that the expression level of C1 was significantly upregulated in the CHD_KDBS group compared with the Normal group (*P* < 0.0001), and was higher than that in the CHD_NKDBS group. The observed difference was statistically significant (*P* < 0.0001), and the result was consistent with the prediction result of the sequencing analysis. The expression level of C4 and C5 in the CHD_KDBS group was significantly lower than that in the Normal group, and was lower than that in the CHD_NKDBS group; the observed difference was statistically significant (*P* < 0.0001). The verification results are shown in Figure 13 (Supplementary Table S7).

## 4. Discussion

In this experiment, high-throughput sequencing technology and bioinformatics analysis methods were used to screen the gene signature profiles of kidney deficiency and blood stasis syndrome in coronary heart disease. The baseline comparison of 60 participants who met the inclusion criteria showed that the HDL levels decreased in the CHD_KDBS group, and the LDL and Cr levels tended to increase. Furthermore, a disparity in the HDL levels was observed between the CHD_NKDBS and the CHD_KDBS groups, indicating that the decline of HDL was an important feature. The differential gene expression profiles of coronary heart disease kidney deficiency and blood stasis syndrome were finally screened by sequencing, and the differences between groups of miRNA and mRNA were determined using stratified cluster analysis. Subsequently, according to the interaction relationship between molecules, an miRNA–mRNA interaction network related to the CHD_KDBS group was constructed, and the functions and signaling pathways involved in the differential genes were excavated.

The complement and coagulation cascade pathway is a complex protein network system that activates the immune system and induces inflammatory responses, phagocyte chemotaxis, and conditioning, which ultimately leads to cytolysis (21–23). The complement system can recruit and activate leukocytes, induce platelet activation and aggregation, and cause thrombosis and inflammation. An abnormal activation and expression of the complement and coagulation cascade in the body can lead to a variety of autoimmune and inflammation-related diseases (24). The complement and coagulation cascade system can play a role in detecting and eliminating pathogens in the body (25), in addition to the clearance of immune complexes, promoting angiogenesis, tissue regeneration, and lipid metabolism (26). The activation of the complement system plays an important role in the treatment of kidney disease, in which C1 antibodies reflect the degree of kidney damage (27). The complement system can also be activated by the coagulation system: in C3 knockout mice, the coagulation system factor thrombin cleaves C5, resulting in a subsequent cascade of activation of C5–9 complement factors (terminal common pathway) (28). Coagulation factors can activate Clqc and mediate the classical pathway activation of complement (29). Complement C5a induces the activated expression of tissue factor (TF), thus promoting the endogenous coagulation pathway (TF-dependent) activation (30, 31).

Several studies have shown that complement and coagulation cascades are associated with cardiovascular-related diseases. A study found that degenerative necrotic tissue cells after myocardial ischemia were enriched in the complement and coagulation cascade pathway, and complement system activation products such as C3, C4, and C5 played a major role in myocardial ischemia-reperfusion inflammatory injury (32). Another study has found that plasma complement and coagulation factor (33) concentrations in patients with cardiomyopathy are significantly higher than those in the Normal groups, and are significantly correlated with the degree of cardiac function (34). Complement may be involved in myocardial tissue inflammation through a variety of pathways, thereby promoting the pathological remodeling process of myocardium (35). It was shown that the complement system is involved in the pathological changes of inflammation and remodeling in patients with end-stage heart failure, and its concentration is significantly related to the severity of symptoms, cardiac function level, BNP concentration, and inflammatory factor concentration in patients with heart failure (36). Similarly, in the myocardial tissue sections of patients with heart failure, the content of complement component deposition is significantly higher than that of normal hearts (37). In addition to myocardial protection, it was suggested that clotting and disruption of the first-line immune system may be factors that lead to psychosis. For example, changes in the immune system can contribute to an increased risk of inflammation, thereby increasing the risk of developing psychiatric disorders (38).

The correlation between the complement–coagulation cascade pathway and kidney deficiency blood stasis syndrome is reflected in the immune system. The complement and coagulation cascade is ubiquitous to maintain innate immune activation (39). A study showed that COVID virus induced the body's complement and coagulation system, and people with a pre-existing coagulopathy condition were found to have a significantly increased risk of dying from the infection (40). A study has suggested that the level of C4 in patients with lung cancer is significantly higher in plasma than in benign patients (41). Another study has found that blood-related exosome components derived from hepatocellular carcinoma are likely to participate in the natural immune response through the complement and coagulation cascade signaling pathway, prevent the spread of cancer cells, and inhibit the proliferation of cancer cells (42). These studies have contributed to a deeper understanding of the intrinsic link between pathway and kidney deficiency and blood stasis syndrome, highlighting the need for additional investigation in future studies on this matter.

## Data Availability

The original contributions presented in the study are included in the article/Supplementary Material. The sequencing raw data have been uploaded in SRA (accession number: PRJNA949421), and the deposited data has been made public (https://www.ncbi.nlm.nih.gov/sra/PRJNA949421). The further inquiries can be directed to the corresponding author.

## References

[1] World Health Statistics 2023: monitoring health for the SDGs, sustainable development goals. Available at: https://www.who.int/data/gho/publications/world-health-statistics.

[2] Chinese Cardiovascular Health and Disease Report Writing Group. Overview of China cardiovascular health and disease report 2021. Chin J Circ. (2022) 37(6):553–78. 10.3969/j.issn.1000-3614.2022.06.001

[3] Han YL Wang XZ. Interventional treatment of unstable angina and non-ST-elevation myocardial infarction. Chin J Pract Intern Med. (2006) 26(8):1131. 10.3969/j.issn.1005-2194.2006.15.005

[4] Meng S Li S Chen H Deng C Meng Z Wang Y. Metabolomics deciphering the potential biomarkers of Hengqing I prescription against vascular dementia. Evid Based Complement Alternat Med. (2022) 2022:1636145. 10.1155/2022/1636145 35399642 PMC8986386

[5] Wang J Li J Yao KW Zhong JB. Study on syndrome elements and indications in coronary heart disease. Tradit Chin Med. (2007) 48(10):920–2. 10.13288/j.11-2166/r.2007.10.029

[6] Wang J Xing YW Chen JX He QY Chen J Xi GC Study on the extraction and combination of angina syndrome in 1069 cases of coronary heart disease by entropy accumulation pile method in complex systems. Chin J Basic Med Tradit Chin Med. (2008) 14(3):211–3. 10.3969/j.issn.1006-3250.2008.03.024

[7] Hinkel R Penzkofer D Zuhlke S Fischer A Husada W Xu QF Inhibition of microRNA-92a protects against ischemia/reperfusion injury in a large-animal model. Circulation. (2013) 128(10):1066–75. 10.1161/CIRCULATIONAHA.113.001904 23897866

[8] Schober A Nazari-Jahantigh M Wei Y Bidzhekov K Gremse F Grommes J MicroRNA-126-5p promotes endothelial proliferation and limits atherosclerosis by suppressing Dlk1. Nat Med. (2014) 20(4):368–76. 10.1038/nm.3487 24584117 PMC4398028

[9] Fichtlscherer S De Rosa S Fox H Schwietz T Fischer A Liebetrau C Circulating microRNAs in patients with coronary artery disease. Circ Res. (2010) 107(5):677–84. 10.1161/CIRCRESAHA.109.215566 20595655

[10] Bao YY. Molecular mechanism of microRNAs in blood stasis of coronary heart disease. Beijing: China Academy of Chinese Medical Sciences (2013).

[11] Golden RJ Chen B Li T Braun J Manjunath H Chen X An Argonaute phosphorylation cycle promotes microRNA-mediated silencing. Nature. (2017) 542(7640):197–202. 10.1038/nature21025 28114302 PMC5302127

[12] Schmiedel JM Klemm SL Zheng Y Sahay A Blüthgen N Marks DS Gene expression. microRNA control of protein expression noise. Science. (2015) 348(6230):128–32. 10.1126/science.aaa1738 25838385

[13] Wu SD Wang J Chen SC Xu JB Zheng Q Yan YF Effect of Xiangdan injection on gene expression of endothelial-derived vasoactive factor in blood stasis of coronary heart disease. J Integr Med. (2004) 02:94–6. 10.3736/jcim20040205 15339465

[14] Yuan ZK Wang LP Huang XP Li J Wang P Yu SR Differential gene screening and functional path analysis related to blood stasis in coronary heart disease. Chin J Integr Med. (2012) 32(10):1313–8. 23163136

[15] Wright RS Anderson JL Adams CD Bridges CR Casey DE Jr Ettinger SM 2011 ACCF/AHA focused update of the guidelines for the management of patients with unstable angina/non-ST-elevation myocardial infarction (updating the 2007 guideline): a report of the American College of Cardiology Foundation/American Heart Association task force on practice guidelines. Circulation. (2011) 123(18):2022–60. 10.1161/CIR.0b013e31820f2f3e 21444889

[16] Wang J Xing YW. Diagnostic criteria for angina syndrome in coronary heart disease. J Tradit Chin Med. (2018) 59(6):539–40. 10.13288/j.11-2166/r.2018.06.023

[17] Cardiology Branch of Chinese Association of Chinese Medicine. Differential diagnostic criteria for the main symptom types of angina pectoris in coronary heart disease. Chin J Integr Tradit West Med. (2018) 38(2):154–5. 10.7661/j.cjim.20171214.330

[18] Shen ZY Wang WJ. Reference standard for false evidence differentiation in Traditional Chinese Medicine. Chin J Integr Med. (1986) 10:598–598.

[19] Liu LC. Screening and verification of miRNA-mRNA in renal deficiency and blood stasis in unstable angina pectoris of coronary heart disease. Beijing: Beijing University of Chinese Medicine (2022).

[20] Wang J Zhang Y Liu YM Yang XC Chen YY Wu GJ Uncovering the protective mechanism of Huoxue Anxin recipe against coronary heart disease by network analysis and experimental validation. Biomed Pharmacother. (2020) 121:109655. 10.1016/j.biopha.2019.109655 31734577

[21] Hoth JJ Wells JD Jones SE Yoza BK McCall CE. Complement mediates a primed inflammatory response after traumatic lung injury. J Trauma Acute Care Surg. (2014) 76(3):601–9. 10.1097/TA.0000000000000129 24553525 PMC4426490

[22] Auger JL Haasken S Binstadt BA. Autoantibody-mediated arthritis in the absence of C3 and activating Fcγ receptors: C5 is activated by the coagulation cascade. Arthritis Res Ther. (2012) 14(6):R269. 10.1186/ar4117 23237573 PMC3674630

[23] Zecher D Cumpelik A Schifferli JA. Erythrocyte-derived microvesicles amplify systemic inflammation by thrombin-dependent activation of complement. Arterioscler Thromb Vasc Biol. (2014) 34(2):313–20. 10.1161/ATVBAHA.113.302378 24311376

[24] Morgan BP Harris CL. Complement, a target for therapy in inflammatory and degenerative diseases. Nat Rev Drug Discov. (2015) 14(12):857–77. 10.1038/nrd4657 26493766 PMC7098197

[25] Ricklin D Lambris JD. Complement in immune and inflammatory disorders: pathophysiological mechanisms. J Immunol. (2013) 190(8):3831–8. 10.4049/jimmunol.1203487 23564577 PMC3623009

[26] Ricklin D Hajishengallis G Yang K Lambris JD. Complement: a key system for immune surveillance and homeostasis. Nat Immunol. (2010) 11(9):785–97. 10.1038/ni.1923 20720586 PMC2924908

[27] Li HX. Research status and prospect of complement system and kidney disease. Chin J Lab Med. (2015) 9:580–2. 10.3760/cma.j.issn.1009-9158.2015.09.002

[28] Huber-Lang M Sarma JV Zetoune FS Rittirsch D Neff TA McGuire SR Generation of C5a in the absence of C3: a new complement activation pathway. Nat Med. (2006) 12(6):682–7. 10.1038/nm1419 16715088

[29] Fruchterman TM Spain DA Wilson MA Harris PD Garrison RN. Complement inhibition prevents gut ischemia and endothelial cell dysfunction after hemorrhage/resuscitation. Surgery. (1998) 124(4):782–92. 10.1067/msy.1998.91489 9781002

[30] Ikeda K Nagasawa K Horiuchi T Tsuru T Nishizaka H Niho Y. C5a induces tissue factor activity on endothelial cells. Thromb Haemost. (1997) 77(2):394–8. 10.1055/s-0038-1655974 9157602

[31] Ritis K Doumas M Mastellos D Anastasia M Stavros G Paola M A novel C5a receptor-tissue factor cross-talk in neutrophils links innate immunity to coagulation pathways. J Immunol. (2006) 177(7):4794–802. 10.4049/jimmunol.177.7.4794 16982920

[32] Bu XM. Pathway-related modules to study the myoprotective effects of sevoflurane and propofol in OPCABG center. Shandong: Shandong University (2019).

[33] Mavroidis M Davos CH Psarras S Aimilia V Nikolaos CA Michalis K Complement system modulation as a target for treatment of arrhythmogenic cardiomyopathy. Basic Res Cardiol. (2015) 110(3):27. 10.1007/s00395-015-0485-6 25851234

[34] Aukrust P Gullestad L Lappegård KT Ueland T Aass H Wikeby L Complement activation in patients with congestive heart failure: effect of high-dose intravenous immunoglobulin treatment. Circulation. (2001) 104(13):1494–500. 10.1161/hc3801.096353 11571242

[35] Iyer A Woodruff TM Wu MC Stylianou C Reid RC Fairlie DP Inhibition of inflammation and fibrosis by a complement C5a receptor antagonist in DOCA-salt hypertensive rats. J Cardiovasc Pharmacol. (2011) 58(5):479–86. 10.1097/FJC.0b013e31822a7a09 21753735

[36] Zwaka TP Manolov D Ozdemir C Marx N Kaya Z Kochs M Complement and dilated cardiomyopathy: a role of sublytic terminal complement complex-induced tumor necrosis factor-alpha synthesis in cardiac myocytes. Am J Pathol. (2002) 161(2):449–57. 10.1016/s0002-9440(10)64201-0 12163370 PMC1850743

[37] Nijmeijer R Krijnen PA Assink J Klaarenbeek MAR Lagrand WK Veerhuis R C-reactive protein and complement depositions in human infarcted myocardium are more extensive in patients with reinfarction or upon treatment with reperfusion. Eur J Clin Invest. (2004) 34(12):803–10. 10.1111/j.1365-2362.2004.01425.x 15606722

[38] Heurich M Föcking M Mongan D Cagney G Cotter DR. Dysregulation of complement and coagulation pathways: emerging mechanisms in the development of psychosis. Mol Psychiatry. (2022) 27(1):127–40. 10.1038/s41380-021-01197-9 34226666 PMC8256396

[39] Wu YB Liu ZL. Congenital immunity and blood coagulation. Thromb Haemost. (2013) 19(1):32–4. 10.3969/j.issn.1009-6213.2013.01.012

[40] Ramlall V Thangaraj PM Meydan C Foox J Butler D Kim J Immune complement and coagulation dysfunction in adverse outcomes of SARS-CoV-2 infection. Nat Med. (2020) 26(10):1609–15. 10.1038/s41591-020-1021-2 32747830 PMC7809634

[41] Zhong RB Li J Yi SQ Wang S Wu XJ Jin HR Analysis of plasma exosome proteome in lung cancer patients. Biotechnol Commun. (2019) 30(1):58–62. 10.3969/j.issn.1009-0002.2019.01.011

[42] Ling YJ Tang YL Ni AN Liao CC Liu MH Liang ZN Hepatocellular carcinoma-derived blood-associated exosome components participate in the innate immunity of tumor tissues through complement and coagulation cascade. Med Princ Pract. (2020) 33(18):2957–60. 10.19381/j.issn.1001-7585.2020.18.001

