# Supplementary material for: Identification and analysis of differential miRNA–mRNA interactions in coronary heart disease: an experimental screening approach
Source: Front Cardiovasc Med. 2023 Oct 30;10:1186297. doi: 10.3389/fcvm.2023.1186297 (PMC10642340; doi:10.3389/fcvm.2023.1186297)
Supplement: Supplementary file 1 [file Datasheet1.docx]

**Supplement 1. Diagnostic criteria for KDBS**

| A | blood stasis syndrome | score | B | kidney deficiency syndrome | score |
| --- | --- | --- | --- | --- | --- |
|  | Chest pain | 4 |  | The waist and knees are sore | 5 |
|  | Tongue is purple and dark or has petechiae | 4 |  | Dizziness, tinnitus | 3 |
|  | Sublingual veins are purple and dark | 3 |  | Hair loss or tooth shaking | 3 |
|  | Complexion is purple and dark | 3 |  | Residual drainage or incontinence after urine | 3 |
|  | Petechiae or ecchymosis on the body | 3 |  | Decreased sexual function | 3 |
|  | Numbness of the limbs | 2 |  | forgetfulness | 3 |
|  | Lips are purple and dark | 2 |  |  |  |
|  | Sluggish pulse | 2 |  |  |  |

Each type of diagnosis must meet at least 1 item A and 1 item in B, and the total points ≥ 8 points can be diagnosed

**Supplement 2. Baseline Data**

|  | **Nomal**  **（n=20）** | **CHD_KDBS**  **（n=20）** | **CHD_NKDBS（n=20）** |
| --- | --- | --- | --- |
| Age | 59.55±1.42 | 62.45±1.13 | 58.75±2.15 |
| Sex（male/%） | 6（30%） | 9（45%） | 7（35%） |
| Smoke | 4（20%） | 6（30%） | 5（25%） |
| Drink | 2（10%） | 4（20%） | 3（15%） |
| Hypertension | 9（45%） | 13（65%） | 11（55%） |
| Diabetes | 4（20%） | 10（50%） | 5（25%） |
| Aspirin | 3（15%） | 7（35%） | 10（50%） |
| Clopidogrel | 1（5%） | 2（10%） | 3（15%） |
| Statins | 6（30%） | 7（35%） | 12（60%） |
| TC（mmol/L） | 4.83±0.17 | 4.23±0.28 | 4.47±0.30 |
| TG（mmol/L） | 1.37±0.65 | 1.72±0.90 | 1.96±1.50 |
| HDL（mmol/L） | 1.57±0.34 | 1.10±0.20^*^ | 1.33±0.26^*△^ |
| LDL（mmol/L） | 2.48±0.73 | 3.05±0.66^*^ | 2.61±0.94 |
| CR（mmol/L） | 61.25±14.23 | 72.00±18.48^*^ | 63.05±14.84 |
| APTT（s） | 29.72±2.19 | 28.24±2.75 | 28.88±4.29 |
| PT（s） | 12.89±0.55 | 12.62±2.88 | 12.74±0.76 |
| D-dimer（mg/L） | 0.27±0.13 | 2.49±0.38^*^ | 0.27±0.08^△^ |

*: P<0.05 compared to normal group; △: P<0.05 compared to CHD_KDBS group

**Supplement 3. RNA sample quality inspection report**

| **sample** | **concentration（μg/μl）** | **A260/280** | **A260/230** | **volume**  **(μl)** | **quantum（μg）** | **RIN** | **result** |
| --- | --- | --- | --- | --- | --- | --- | --- |
| A1 | 0.5062 | 1.92 | 1.65 | 10 | 5.06 | 7.4 | qualified |
| A2 | 0.4374 | 1.96 | 1.52 | 10 | 4.37 | 7.4 | qualified |
| A3 | 0.4129 | 1.95 | 1.07 | 10 | 4.13 | 9.0 | qualified |
| A4 | 0.2180 | 1.89 | 1.32 | 10 | 2.18 | 6.8 | qualified |
| A5 | 0.3130 | 1.92 | 1.07 | 45 | 14.09 | 9.6 | qualified |
| B1 | 0.3753 | 1.99 | 1.68 | 55 | 20.64 | 9.1 | qualified |
| B2 | 0.1774 | 1.94 | 1.62 | 55 | 9.76 | 9.3 | qualified |
| B3 | 0.2835 | 1.96 | 1.48 | 55 | 15.59 | 8.4 | qualified |
| B4 | 0.3613 | 1.98 | 1.86 | 55 | 19.87 | 7.4 | qualified |
| B5 | 0.2583 | 1.97 | 1.54 | 55 | 14.21 | 9.3 | qualified |
| C1 | 0.4391 | 1.97 | 1.60 | 25 | 10.98 | 8.3 | qualified |
| C2 | 0.4546 | 1.99 | 1.87 | 50 | 22.73 | 8.4 | qualified |
| C3 | 0.2706 | 1.93 | 1.70 | 25 | 6.77 | 7.5 | qualified |
| C4 | 0.1528 | 1.93 | 1.69 | 10 | 1.53 | 8.6 | qualified |
| C5 | 0.3450 | 1.98 | 1.62 | 25 | 8.63 | 8.6 | qualified |

A：Nomal group；B：CHD_KDBS group；C：CHD_NKDBS group

**Supplement 4. Data statistics after quality preprocessing of original sequencing results**

| **Sample** | **CleanReads** | **CleanBases** | **Q30** | **GC** |
| --- | --- | --- | --- | --- |
| A1 | 101.02M | 14.60G | 91.70% | 46.27% |
| A2 | 97.75M | 14.04G | 91.74% | 45.61% |
| A3 | 93.95M | 13.30G | 90.67% | 46.35% |
| A4 | 93.34M | 13.35G | 91.07% | 45.98% |
| A5 | 99.12M | 14.15G | 90.95% | 46.04% |
| B1 | 99.61M | 14.29G | 90.93% | 45.40% |
| B2 | 95.88M | 13.61G | 89.47% | 45.75% |
| B3 | 99.73M | 14.29G | 90.20% | 45.40% |
| B4 | 101.03M | 14.54G | 90.82% | 45.87% |
| B5 | 92.75M | 13.25G | 90.70% | 45.28% |
| C1 | 94.57M | 13.50G | 89.42% | 45.02% |
| C2 | 99.49M | 14.28G | 88.98% | 45.63% |
| C3 | 96.15M | 13.74G | 88.96% | 45.23% |
| C4 | 92.65M | 13.23G | 89.12% | 45.55% |
| C5 | 91.20M | 12.83G | 86.66% | 44.75% |

A：Nomal group；B：CHD_KDBS group；C：CHD_NKDBS group

**Supplement 5 miRNA-mRNA co-regulatory network**

| **miRNA** | **mirnaLogFC** | **mRNA** | **mrnaLogFC** |
| --- | --- | --- | --- |
| hsa-miR-4433b-3p | 1.169469291 | EGR3 | -1.26964949 |
|  |  | TTN | -0.631439912 |
|  |  | TP53INP2 | -0.753112796 |
|  |  | SIK1 | -0.924467602 |
|  |  | SRGAP3 | -0.706934706 |
| hsa-miR-6852-5p | 0.71271115 | EGR3 | -1.26964949 |
|  |  | COL6A1 | -0.692233065 |
|  |  | CDHR3 | -0.630019829 |
| hsa-miR-1180-3p | -1.030955267 | PTPRS | 0.712111798 |
|  |  | TNS3 | 0.625472476 |
| hsa-miR-3150b-3p | 0.825502571 | NOG | -1.307236465 |
|  |  | ARL5B | -0.631166863 |
| hsa-miR-324-5p | -1.035437012 | SERPINF1 | 0.706035345 |
|  |  | MYO1E | 0.686916381 |
| hsa-miR-363-5p | -0.857302788 | MYEF2 | 0.823413371 |
|  |  | TRIM7 | 0.813936508 |
| hsa-miR-3928-3p | 0.719922265 | CUBN | -0.72994446 |
| hsa-miR-4685-3p | -0.825892299 | COL10A1 | 2.092339204 |
|  |  | SH3PXD2B | 0.841042377 |
|  |  | C1QC | 1.513530591 |
|  |  | RUSC2 | 0.617701218 |
|  |  | ARPIN | 0.667202052 |
|  |  | CEBPA | 0.752750486 |
|  |  | KCND3 | 0.789811586 |
|  |  | SH3TC2 | 0.828038936 |
|  |  | KCTD12 | 0.725223564 |
|  |  | CMTM4 | 0.705421651 |
|  |  | ZNF385D | 1.132590822 |
|  |  | RHD | 1.438463023 |
| hsa-miR-5001-3p | -0.755187536 | IFITM10 | 0.592263505 |
|  |  | CCDC103 | 1.354434561 |
|  |  | MYO18B | 2.214102922 |
|  |  | KIF3C | 1.088942329 |
|  |  | SEC14L5 | 1.380928274 |
|  |  | RAB6B | 1.082159813 |
| hsa-miR-5187-5p | 0.937532615 | XKR6 | -0.587579806 |
| hsa-miR-6769b-3p | 1.568423652 | PTCH2 | -0.728431475 |
| hsa-miR-6815-5p | -1.57415681 | CES1 | 1.838350086 |
|  |  | C1orf127 | 1.183730421 |
|  |  | ARG2 | 0.81584675 |
|  |  | PTPRN2 | 1.1538528 |
|  |  | MAP1A | 0.990913578 |
|  |  | TMEM144 | 1.075916125 |
|  |  | DOCK1 | 0.826225106 |
| novel1804_mature | -2.315394586 | NRP1 | 0.777721916 |
|  |  | CD276 | 2.866510485 |
|  |  | CYFIP1 | 0.629980459 |
|  |  | FHL1 | 0.75645075 |
|  |  | PLEKHN1 | 1.001297822 |
|  |  | COL8A2 | 1.057721973 |
|  |  | STK32B | 1.252897131 |
|  |  | SERPINF1 | 0.706035345 |
|  |  | FCN1 | 0.772304208 |
|  |  | TMCC2 | 0.663279892 |
|  |  | RASL10B | 1.172542047 |
|  |  | CMTM4 | 0.705421651 |
| novel1_star | 1.020111134 | KANK1 | -1.160078887 |
| novel218_mature | -1.57375429 | HBE1 | 2.864680823 |
| novel49_mature | 1.070321461 | C4B | -1.029038098 |
| hsa-miR-1273h-3p | 0.655067505 | MAFF | -0.69702935 |
|  |  | GOLGA6L10 | -0.92014139 |
|  |  | TTN | -0.631439912 |
| novel1232_mature | -1.643608121 | IER5L | 0.847099109 |

**Supplement 6 Transcription expression level**

|  | **n** | **Nomal** | **CHD_KDBS** | **CHD_NKDBS** |
| --- | --- | --- | --- | --- |
| miR-4685-3p | 15 | 0.00959±0.00191 | 0.00102±0.00029 | 0.00231±0.00118 |
| C1 | 15 | 0.00026±0.00006 | 0.00457±0.00098 | 0.00344±0.00158 |
| C4 | 15 | 0.00850±0.00448 | 0.00674±0.00328 | 0.00744±0.00312 |
| C5 | 15 | 0.00071±0.00015 | 0.00036±0.00005 | 0.00049±0.00018 |

*****: P=0.0001 *: P=0.0190**

**Supplement 7 Protein expression level**

|  | **n** | **Nomal** | **CHD_KDBS** | **CHD_NKDBS** |
| --- | --- | --- | --- | --- |
| C1 | 15 | 99.03±12.04 | 231.68±5.11 | 112.24±8.61 |
| C4B | 15 | 650.86±155.15 | 390.04±47.86 | 822.06±56.17 |
| C5 | 15 | 1050.83±102.84 | 514.88±19.74 | 883.04±88.54 |

****: P＜0.0001 **: P=0.0011
